# Anatomic variant of the internal jugular vein and its importance in vascular access for hemodialysis

**DOI:** 10.1590/1677-5449.190014

**Published:** 2019-10-23

**Authors:** Aline Ioshie Akamine Asari, Ricardo André Viana Barros, Marcos Aurélio Perciano Borges

**Affiliations:** 1 Hospital de Base do Distrito Federal – HBDF, Unidade de Cirurgia Vascular, Brasília, DF, Brasil.

**Keywords:** renal insufficiency, renal dialysis, anatomic variation, embryology, ultrasonography

## Abstract

The right internal jugular vein is considered the best route for vascular access, because of low complication rates and satisfactory flow during hemodialysis, due to its straight route to the right atrium. This paper reports the identification, prior to puncture, of an anatomic variant position of the internal jugular vein in relation to the common carotid artery. The benefit of this prior identification is highlighted, emphasizing the importance of performing vascular Doppler ultrasound rather than using only external anatomical observation for puncture of the internal jugular vein.

## INTRODUCTION

The internal jugular vein (IJV) is an extremely important option for central venous access for many different purposes, including hemodialysis (HD), for example. The IJV and the other cephalic veins undergo a complicated process of embryonic development, with countless opportunities for abnormal development, regressions, or anastomoses, creating many different anatomic variants.^1^

Before puncturing the IJV, it is imperative to identify the anatomic structures of the specific patient involved, in order to avert complications such as inadvertent arterial puncture, pneumothorax, hemothorax, chylothorax, hematoma, brachial plexus injury, gaseous emboli, catheter knotting, arrhythmia, arteriovenous fistula, ruptured right atrium, vocal chord paralysis, and severe respiratory obstruction. These complications all contribute to increased morbidity, extended hospital stays, and increased hospital costs.^2^^-^ 5

Variants in the positions of the vessels commonly employed for catheter access comprise a pitfall that increases complication rates.^2^^,^ 3 This is why detecting anatomic variants of the IJV using vascular ultrasound with Doppler (USD) results in higher insertion success rates, as emphatically recommended by the National Kidney Foundation (NKF KDOQI)™ since 1997.^6^

This article describes identification of an anatomic variant in the position of the IJV in relation to the common carotid artery (CCA) using USD and discusses, with a review of the literature, the benefits of employing this practice before puncturing the IJV.

## CASE DESCRIPTION

A 76-year-old male patient with chronic renal failure with indications for HD underwent USD of the cervical and proximal veins of the upper limbs for analysis and selection of the ideal vein for puncture and insertion of the HD catheter.

**Figure 1:**
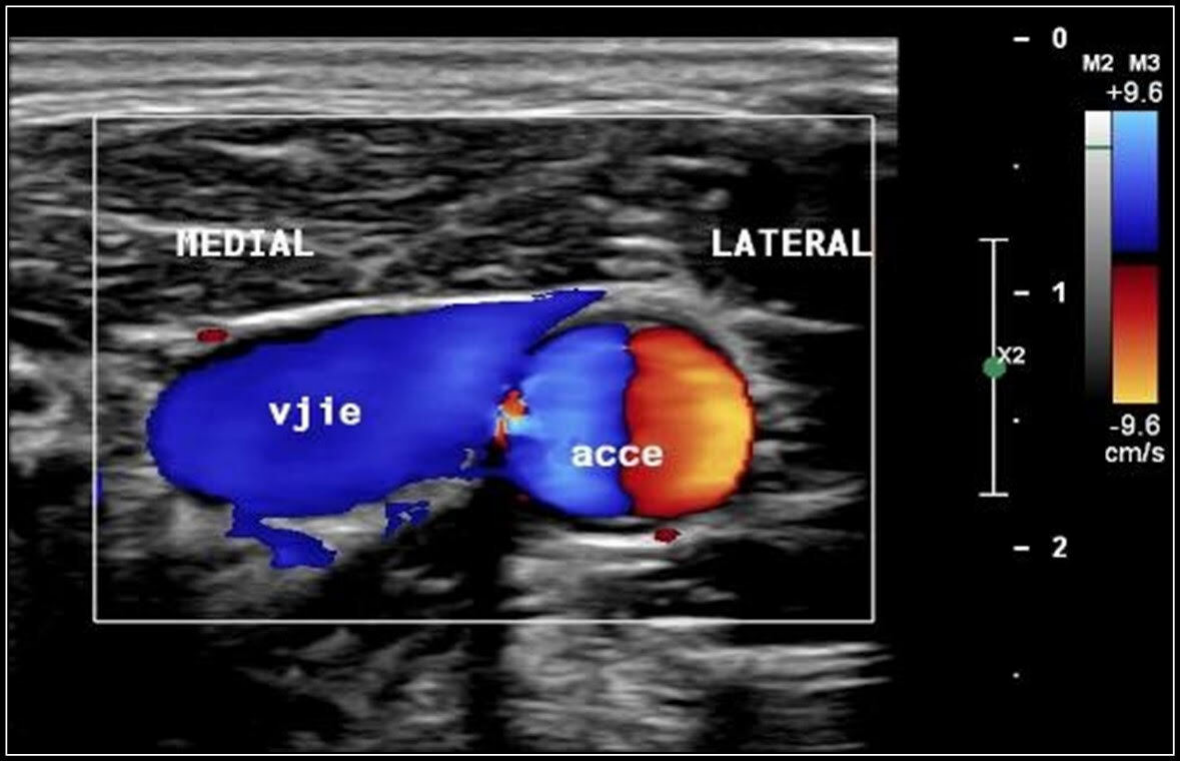
Vascular ultrasound with Doppler (mode B) of the left cervical area, showing the position of the internal jugular vein medial to the path of the common carotid artery (at the same level and at a distance < 10 mm).

**Figure 2:**
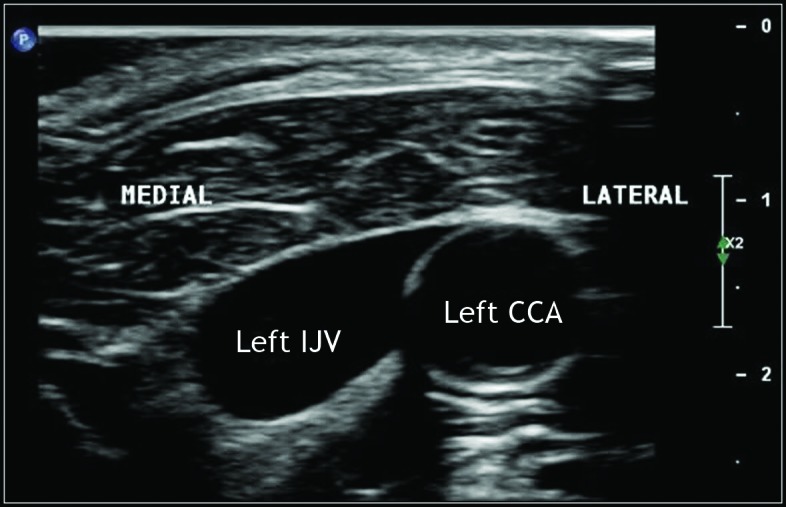
Vascular ultrasound with color Doppler of the left cervical area, showing the position of the internal jugular vein (IJV) medial to the path of the common carotid artery (CCA).

The ultrasound scan (Philips© Affiniti 50) with linear transducer (L/12-3 MHz), detected the right IJV in the expected anatomic position, i.e. superficial and lateral to the right CCA, but showed an anatomic variant of the left IJV, which had an adequate diameter but was located medially and at the same level as the path taken by the left CCA (Figures 1 and 2).

## DISCUSSION

The IJV is responsible for drainage of the majority of the structures in the cranial cavity and the deeper portions of the face and neck.^7^ It originates at the base of the skull, in the posterior compartment of the jugular foramen, running distally in the vertical direction within the carotid sheath. Its route runs lateral to the internal carotid branch and the CCA.^7^

The IJV is the largest vein in the head and neck region and as the continuation of the sigmoid sinus it drains the intracranial, orbital, and superficial structures of the face and neck.^8^^,^ 9

During development of the cranium, the first vessel identified is the ventral pharyngeal vein, which drains the major part of the mandible bone and the hyoid arch to the common cardinal vein. As the neck grows longitudinally, its drainage derives the cranial portion of the precardinal veins, also known as the anterior cardinal veins. These, in turn, will become the right and left internal jugular veins.^8^^,^ 10

The medical literature contains a small number of studies investigating anatomic variants of the IJV, primarily with fetal samples. Pillay et al.^9^ described the results of dissections of 80 fetuses, demonstrating that 1/80 (1%) specimens exhibited a Y-shaped IJV; 2/80 (3%) specimens had an IJV divided in two that were tributaries of the external jugular vein superiorly and inferiorly.

Mumtaz and Singh^11^ conducted a review analyzing 1,197 cases, reporting the following IJV variants: bifurcation (4 cases), duplication (14), fenestration (16), trifurcation (1) and posterior tributary (5). They did not report variants involving a jugular vein lateral to the CCA.

In the majority of clinical conditions, access to the IJV is easily obtained using external anatomic references, which is a technique that is considered safe and easy, with success rates of 85 to 99%, and is therefore used by physicians working in several different specialties. However, difficulties can occur in a significant percentage of cases. The rate of puncture failure with this access varies from 7 to 19.4%, depending on the experience of the operator.^5^^,^ 12

A study conducted in July 2016 showed that Brazil had an estimated 122,825 patients on dialysis, 92% of whom were on HD and 8% of whom were on peritoneal dialysis. A central venous catheter is used in 20.5% of cases.^13^

Complications related to central venous puncture for HD catheter fitting can be divided into three categories: mechanical, thromboembolic, and infectious.

Anatomic variants are potential causes of mechanical complications, identified in 30% of cases, according to Gallieni.^12^ In the Western population, anatomic variant rates exceeding 12% of patients have been reported, mostly small venous diameters. The IJV is generally at least twice the size of the CCA, at around 9.1 to 10.2 mm.

Anatomic assessment of the IJV in patients with uremia can be performed using USD to analyze: vein diameter, considering ≥ 5 mm adequate; normal location in relation to the CCA, i.e., superficial and lateral with a maximum distance of 10 mm between them; and unilateral or bilateral occurrence.^2^

Doppler ultrasound increases the safety and efficacy of the technique for percutaneous cannulation of the IJV and can guide the puncture needle and rapidly establish reasons for failure, reducing risk and patient discomfort. The technique is useful when an anatomic reference is distorted or cannot be located, as can happen in patients with short necks, obesity, or a history of previous punctures, or who have undergone radiotherapy and surgery in the neck area.^5^

In a study by Denys and Uretsky^5^ that analyzed 200 patients, 5.5% had an IJV that did not correspond to the site predicted by external markings. In 3%, the IJV was in the expected position, but with a small caliber, making it a difficult target. In 8.5% of cases, the IJV anatomy was sufficiently aberrant to complicate access via blind methods. These findings could contribute to the 10% failure rate even with experienced operators when the puncture by anatomic observation technique is used. The risk increases the more medial the puncture site.

**Figure 3:**
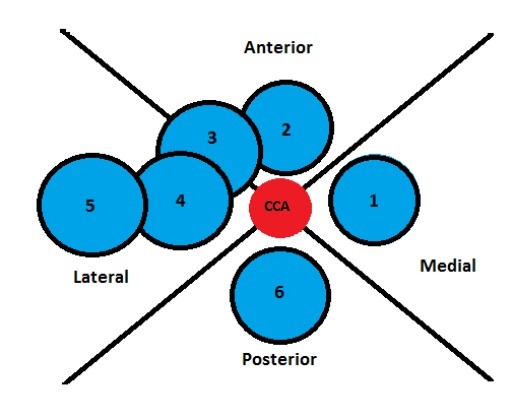
Diagram illustrating the relationship between the left internal jugular vein (IJV) and the left common carotid artery (CCA), shown in the center in red. 1. IJV medial of the CCA, incidence of 0-5.5% of cases; 2. IJV anterior of the CCA, 0-16%; 3. IJV anterolateral of the CCA, 9-92%; 4. IJV lateral of the CCA, 0-84%; 5. IJV more than 10 mm lateral of the CCA, 0-4%; 6. IJV posterior of the CCA, 0-9%.

In a study by Prasad et al.,^14^ the IJV was in the lateral and anterolateral position in 86.66% of cases on the right and in 85% on the left, which is the safe position for anatomic reference-based puncture. In a survey conducted by Maecken and Grau,^15^ the rate of an IJV medial of the CCA found in studies that used ultrasound varied from 0 to 5.5%, as illustrated in Figure 3. Lim et al.^16^ analyzed tomographic images, identifying an IJV medial of the CCA in 1.1% of cases, confirming the rarity of finding the vein completely medial of the artery.

Figure 3 illustrates the possible anatomic variants of the IJV, on the left in this case, as in our case report. It also shows the frequencies of each finding in the review performed by Maecken and Grau.^15^ Cases in which the IJV was not located or was thrombosed accounted for 18% of the total.

The most recent KDOQI guidelines set an objective of 65% autogenous accesses for dialysis patients, with a rate of catheter use of less than 10% in the absence of a satisfactory autogenous access. Patency rates for prosthetic accesses should exceed 2 years, with a thrombosis rate of less than 0.5 episodes/patient year, and an infection rate lower than 10% over the service life of the access.^17^

In view of the above, it is clear how important it is to use USD to locate and to judge the feasibility of puncture of the IJV, or any other central vein, in patients with a need for HD access, thereby preventing complications that could be caused by anatomic variations.
