# Supplementary material for: Anatomic variant of the internal jugular vein and its importance in vascular access for hemodialysis
Source: J Vasc Bras. 2019 Oct 23;18:e20190014. doi: 10.1590/1677-5449.190014 (PMC6822959; doi:10.1590/1677-5449.190014)
Supplement: Translation

# Translation: Variação anatômica da veia jugular interna e sua importância no acesso vascular para hemodiálise

**Authors:** Aline Ioshie Akamine Asari, Ricardo André Viana Barros, Marcos Aurélio Perciano Borges

**Affiliations:** 1 Hospital de Base do Distrito Federal – HBDF, Unidade de Cirurgia Vascular, Brasília, DF, Brasil.

**Keywords:** insuficiência renal, diálise renal, variação anatômica, embriologia, ultrassonografia

## Abstract

A veia jugular interna direita é considerada a melhor via para um acesso vascular, devido a baixas taxas de complicação e ao fornecimento de fluxo satisfatório durante a hemodiálise dado seu trajeto mais retilíneo para o átrio direito. O presente estudo relata a identificação, prévia à punção, de uma variação anatômica da posição da veia jugular interna em relação à artéria carótida comum. Destaca-se o benefício dessa identificação prévia, enfatizando-se a importância de se realizar a ultrassonografia vascular com Doppler em detrimento de utilizar somente reparos anatômicos externos para a punção da veia jugular interna.

## INTRODUÇÃO

A veia jugular interna (VJI) é de suma importância como acesso venoso central para diversos propósitos, por exemplo a hemodiálise (HD). A VJI e as outras veias cefálicas apresentam uma história complicada de evolução embrionária, com inúmeras oportunidades para desenvolvimento anormal, regressões ou anastomoses, gerando diversas variações anatômicas^1^.

Antes de se realizar a punção da VJI, é imperativo o reconhecimento das estruturas anatômicas específicas do paciente visando evitar complicações, tais como: punção arterial inadvertida, pneumotórax, hemotórax, quilotórax, hematoma, lesão de plexo braquial, embolia gasosa, nó do cateter, arritmia, fístula arteriovenosa, ruptura do átrio direito, paralisia de corda vocal e obstrução respiratória severa. Todas essas complicações contribuem para o aumento da morbidade, o prolongamento da internação e o aumento dos custos hospitalares^2^^-^ 5.

As variações de posicionamento dos vasos comumente utilizados para fornecer acesso aos cateteres são uma armadilha que aumenta os índices de complicações^2^^,^ 3. Por isso, a detecção das variações anatômicas da VJI por meio da ultrassonografia vascular com Doppler (USD) resulta em taxas maiores de sucesso do implante, sendo amplamente recomendada pela National Kidney Foundation (NKF KDOQI)™ desde 1997^6^.

Neste trabalho é relatada a identificação de uma variação anatômica da posição da VJI em relação à artéria carótida comum (ACC) por meio de USD e é discutido, por meio de revisão de literatura, o benefício dessa prática antes de se realizar a punção da VJI.

## DESCRIÇÃO DO CASO

Paciente masculino, 76 anos, portador de insuficiência renal crônica com indicação de HD, submetido a USD das veias cervicais e proximais dos membros superiores para análise e seleção da veia ideal para a punção e implante do cateter de HD.

O exame ultrassonográfico (Philips © Affiniti 50), transdutor linear (L/12-3 MHz), detectou a VJI direita em posição anatômica habitual, ou seja, superficial e lateral à ACC direita; porém, identificou uma variação anatômica na VJI esquerda, que apresentava com diâmetro adequado mas estava posicionada medialmente e no mesmo nível do trajeto da ACC esquerda (Figuras 1 e 2).

**Figura 1:** Ultrassonografia vascular com Doppler (modo B) cervical à esquerda demonstrando o posicionamento da veia jugular interna medial ao trajeto da artéria carótida comum (mesmo nível e a uma distância < 10 mm).

**Figura 2:** Ultrassonografia vascular com Doppler colorido cervical à esquerda demonstrando o posicionamento da veia jugular interna medial ao trajeto da artéria carótida comum.

## DISCUSSÃO

A VJI é responsável pela drenagem da maior parte das estruturas da cavidade craniana e das porções profundas da face e do pescoço^7^. Origina-se na base do crânio, no compartimento posterior do forâmen jugular, seguindo distalmente em sentido vertical na bainha carotídea. Durante seu curso, localiza-se lateralmente ao ramo carotídeo interno e à ACC^7^.

A VJI é a maior veia da região da cabeça e do pescoço e sua continuação do seio sigmoide drena as estruturas intracranianas, orbitais e superficiais da face e do pescoço^8^^,^ 9.

Com o desenvolvimento do crânio, o primeiro vaso identificado é a veia faríngea ventral, que drena a maior parte do osso da mandíbula e do arco hioide para a veia cardinal comum. À medida que o pescoço cresce longitudinalmente, sua drenagem deriva para a porção cranial das veias pré-cardinais, também denominadas de veias cardinais anteriores. Essas, por sua vez, se tornarão as veias jugulares internas direita e esquerda^8^^,^ 10.

A literatura médica é escassa em estudos que avaliem as variações anatômicas da VJI, principalmente com amostra em fetos. Pillay et al.^9^ descreveram os resultados da dissecção de 80 fetos, demonstrando que 1/80 (1%) dos espécimes apresentavam a VJI em formato de “Y”; 2/80 (3%) dos espécimes apresentavam a VJI dividida em duas e sendo tributária da veia jugular externa superior e inferiormente.

Mumtaz e Singh^11^, em uma revisão que analisou 1.197 casos, cita as seguintes variações da VJI: bifurcação (4 casos), duplicação (14), fenestração (16), trifurcação (1) e tributária posterior (5). Contudo, não se verifica relato de variações quanto à lateralidade da veia jugular em relação à ACC.

Na maioria das condições clínicas, o acesso à VJI é facilmente obtido utilizando as referências anatômicas externas, técnica considerada segura e fácil, com taxa de sucesso de 85% a 99%, sendo por isso utilizada por médicos de várias especialidades. Entretanto, pode ocorrer dificuldade em um percentual significativo dos casos. A taxa de falha de punção desse acesso varia de 7% a 19,4%, a depender da experiência do operador^5^^,^ 12.

Um estudo realizado em julho de 2016 mostra que o Brasil contabiliza um número total estimado de 122.825 pacientes em diálise, sendo que 92% deles estão em HD e 8% em diálise peritoneal. O cateter venoso central é utilizado em 20,5% dos casos^13^.

As complicações de punção venosa central para o implante de cateter de HD podem ser divididas em três categorias: mecânicas, tromboembólicas e infecciosas.

As variações anatômicas são causas potenciais de complicações mecânicas, sendo identificadas em 30% dos casos, segundo Gallieni^12^. Na população ocidental, têm sido reportadas taxas superiores a 12% de pacientes com variações anatômicas, principalmente diâmetros venosos reduzidos. Habitualmente, a VJI apresenta pelo menos o dobro do tamanho da ACC, com cerca de 9,1 mm a 10,2 mm.

A avaliação anatômica da VJI em pacientes urêmicos pode ser realizada pela USD, na qual são analisados: diâmetro da veia, considerando-se adequado ≥ 5 mm; localização habitual em relação à ACC, ou seja, superficial e lateral com distância máxima entre elas de 10 mm; ocorrência uni ou bilateral^2^.

A USD adiciona segurança e eficiência à técnica da canulação percutânea da VJI, podendo guiar a agulha de punção e rapidamente estabelecer a sua razão de falha, diminuindo o risco e o desconforto do paciente. Essa técnica é boa quando a referência anatômica está distorcida ou não evidente, como ocorre em pacientes com pescoço curto, obesos, com histórico de punções prévias ou submetidos a radioterapia e cirurgia na região do pescoço^5^.

No estudo de Denys e Uretsky^5^, que analisou 200 pacientes, 5,5% deles apresentavam a VJI não correspondente ao local predito pela marcação externa. Em 3%, a VJI estava na posição esperada, porém com calibre reduzido, tornando-se um alvo difícil. Em 8,5% dos casos, a anatomia da VJI era suficientemente aberrante para complicar o acesso por meio de método às cegas. Esses achados podem contribuir para a taxa de falha de 10% mesmo entre operadores experientes, quando a técnica de punção por reparos anatômicos é empregada. O risco aumenta quanto mais medial for a punção.

No estudo de Prasad et al.^14^, a VJI encontrava-se em posição lateral e anterolateral em 86,66% dos casos à direita e em 85% à esquerda, correspondendo à posição segura para punção baseada em referenciais anatômicos. Em um levantamento realizado por Maecken e Grau^15^, o achado de VJI medial à ACC em estudos que usavam o exame ultrassonográfico variou de 0 a 5,5%, conforme ilustrado na Figura 3. Lim et al.^16^, ao analisarem imagens tomográficas, identificaram VJI medial à ACC em 1,1% dos casos, corroborando a raridade do achado da veia completamente medial à artéria.

**Figura 3:** Representação da relação da veia jugular interna (VJI) esquerda em relação à artéria carótida comum (ACC) esquerda, esta representada ao centro em vermelho. 1. VJI medial à ACC, incidência de 0-5,5% dos casos; 2. VJI anterior à ACC, 0-16%; 3. VJI anterolateral à ACC, 9-92%; 4. VJI lateral à ACC, 0-84%; 5. VJI a mais de 10 mm lateral à ACC, 0-4%; 6. VJI posterior à ACC, 0-9%.

A Figura 3 ilustra as possíveis variações anatômicas da VJI, no caso representada à esquerda como em nosso relato de caso, bem como a frequência de seus achados conforme revisão realizada por Maecken e Grau^15^. Os casos em que a VJI não era visualizada ou apresentava-se trombosada chegavam a até 18% do total.

As diretrizes KDOQI mais recentes preconizam como objetivo o emprego de 65% de acessos autógenos entre os pacientes de diálise, com taxa de utilização de cateteres menor que 10% na ausência de um acesso autógeno satisfatório. Os índices de permeabilidade para acessos protéticos devem ser superiores a 2 anos, com taxa de trombose inferior a 0,5 episódio/paciente ao ano e taxa de infecção inferior a 10% ao longo da vida útil do acesso^17^.

A partir do exposto, verifica-se a importância do uso de USD para a localização e viabilidade da punção da VJI ou de qualquer outra veia central em pacientes com necessidade de acesso para HD, prevenindo-se complicações que podem advir de variações anatômicas.
